# Clinical Manifestations and Myositis-Specific Autoantibodies Associated with Physical Dysfunction after Treatment in Polymyositis and Dermatomyositis: An Observational Study of Physical Dysfunction with Myositis in Japan

**DOI:** 10.1155/2016/9163201

**Published:** 2016-01-26

**Authors:** Hidenaga Kawasumi, Takahisa Gono, Yasushi Kawaguchi, Masataka Kuwana, Hirotaka Kaneko, Yasuhiro Katsumata, Sayuri Kataoka, Masanori Hanaoka, Hisashi Yamanaka

**Affiliations:** ^1^Institute of Rheumatology, Tokyo Women's Medical University, 10-22 Kawada-cho, Shinjuku-ku, Tokyo 162-0054, Japan; ^2^Department of Allergy and Rheumatology, Nippon Medical School Graduate School of Medicine, 1-1-5 Sendagi, Bunkyo-ku, Tokyo 113-8603, Japan

## Abstract

*Objective.* The physical function of PM/DM patients after remission induction therapy remains unknown adequately. The aim of our study was to evaluate the present status of physical dysfunction and to clarify the clinical manifestations and myositis-specific autoantibodies (MSAs) associated with physical dysfunction after treatment in PM/DM.* Methods.* We obtained clinical data including the age at disease onset, gender, disease duration, laboratory data prior to initial treatment, and the specific treatment administered. We evaluated disease activity and physical dysfunction after treatment using the core set provided by the International Myositis Assessment and Clinical Studies Group.* Results.* 57% of the 77 enrolled patients with PM/DM had troubles in daily living after treatment. At the enrolment, disease activity evaluated by physicians was only revealed in 20% of patients. In a multivariate analysis, the age at disease onset, female gender, and CK levels before treatment were significantly associated with the severity of physical dysfunction after treatment. Anti-SRP positivity was associated with more severe physical dysfunction after treatment than anti-ARS or anti-MDA5.* Conclusions.* Half of the PM/DM patients showed physical dysfunction after treatment. Age at disease onset, gender, CK level before treatment, and anti-SRP were significant predictors associated with physical dysfunction after treatment in PM/DM.

## 1. Introduction

Polymyositis (PM) and dermatomyositis (DM) are idiopathic inflammatory myopathies that occasionally present with extramuscular lesions such as interstitial lung disease (ILD) [1, 2], cardiomyopathy [3], and malignancy [4]. Some PM/DM patients still suffer from muscle weakness and physical dysfunction after remission induction therapies [5]. As a result, these patients have trouble with daily living even after their disease activity is adequately controlled. Sustained physical dysfunction after treatment may be associated with the PM/DM disease duration, irreversible muscle damage, and the adverse effects of corticosteroids such as myopathy, vertebral compression fracture, and avascular necrosis [5, 6].

Recent PM/DM therapeutic strategies have improved the overall survival prognosis of patients [6–8]. In addition, several myositis-specific autoantibodies (MSAs) have been identified and are useful for predicting clinical manifestations, treatment outcomes, and vital prognoses [9–11]. For example, patients with anti-Mi-2 antibodies more commonly develop DM, and these patients are less likely to develop ILD or malignancy [12–14]. Moreover, the treatment outcomes of anti-Mi-2-positive patients are relatively better than those with other autoantibodies. In contrast, patients with anti-signal recognition particle (SRP) antibodies often develop necrotising myopathy, which is refractory to corticosteroid therapy, and a tapering dosage of corticosteroids often causes a recurrence of the myositis [15–21]. However, the physical function outcomes of PM/DM patients after remission remain poorly characterised. Moreover, the predictive factors of physical dysfunction following treatment among PM/DM patients remain unknown.

In the present study, we evaluated the present status of physical dysfunction in PM/DM outpatients after treatment. Moreover, we identified clinical manifestations and MSAs that are associated with physical dysfunction after treatment.

## 2. Patients and Methods

### 2.1. Patients

Among the PM/DM outpatients who regularly visited our hospital from August to October 2013, informed consent was obtained from seventy-seven outpatients. These 77 PM/DM patients were enrolled in the present study. Some of the included patients also had clinically amyopathic DM (CADM). All of these patients were previously admitted to our hospital to receive remission induction therapy for PM/DM. At the time of admission, all patients had not received remission induction therapy yet. The diagnoses of PM, DM, or CADM were made based on the criteria of Bohan and Peter [22] or those of Sontheimer [23].

We obtained clinical data from the medical records of all the enrolled patients. These clinical data included the age at disease onset, gender, disease duration, laboratory data prior to initial treatment (e.g., plasma creatinine kinase (CK), lactate dehydrogenase (LDH), and C-reactive protein (CRP) levels), extramuscular lesions (ILD, cardiomyopathy, and malignant disease), the specific treatment administered, and the occurrence of relapse. This study was approved by the Ethical Committee of Tokyo Women's Medical University according to the Declaration of Helsinki.

### 2.2. Evaluation of Serum Myositis-Specific Autoantibodies and Myositis-Associated Autoantibodies

Serum samples were obtained from 67 patients on admission and were stored at −80°C. In the other 10 patients, the serum samples were not stored and could not be evaluated. We evaluated the positivity of MSAs and myositis-associated autoantibodies (MAAs). Anti-aminoacyl-tRNA synthetase (anti-ARS), anti-SRP, anti-Ku, and anti-SS-A antibodies were evaluated using an immunoprecipitation assay. Anti-melanoma differentiation-associated gene 5 (anti-MDA5) antibodies were measured with an enzyme-linked immunosorbent assay. Anti-transcriptional intermediary factor 1-*γ* (anti-TIF1-*γ*), anti-Mi-2, and anti-nuclear matrix protein-2 (anti-NXP-2) antibodies were detected using western blot. Anti-U1RNP antibodies were measured using an Ouchterlony double immunodiffusion assay.

### 2.3. Evaluation of Disease Activity and Physical Function

We evaluated the disease activity, muscle strength, and physical function of each patient one time after treatment from August to October 2013 in our outpatient ward. Disease activity was evaluated using the myositis disease activity core set provided by the International Myositis Assessment and Clinical Studies (IMACS) Group [24, 25]. The disease activities were evaluated by the patients (the Patient Global Assessment (PGA)) and the physicians (the Evaluator's Global Assessment (EGA)) on a 0–100-point scale. In the present study, we defined the presence of disease activity as PGA or EGA scores greater than 10 points. Muscle strength was evaluated using the manual muscle test for eight muscles (MMT8) using a 0–10-point scale [26]. We evaluated the severity of physical function using the Japanese version of the Health Assessment Questionnaire Disability Index (J-HAQ-DI) [27]. The Stanford HAQ is a self-report questionnaire assessing physical function pertaining to the activities of daily living [28]. The J-HAQ was adapted directly from the Stanford HAQ and was first published in 2003 [27]; this instrument was translated into Japanese with culturally appropriate modifications. Although the J-HAQ was originally developed for use in rheumatoid arthritis, it has been applied to a variety of rheumatic conditions in Japan. In the present study, physical dysfunction was defined as a J-HAQ-DI score greater than 0.5 [29].

### 2.4. Statistical Analysis

Statistical analyses were performed using the Mann-Whitney *U* test to compare median values. The correlation coefficients were established by Spearman's correlation coefficient. The multivariate analysis was performed using multiple regression analysis. The data were analysed using JMP^®^ software (SAS Institute, NC, USA). *P* values <0.05 indicated statistical significance.

## 3. Results

### 3.1. Clinical Characteristics of the Enrolled Patients

As shown in Table 1, 77 patients were enrolled in this study. The median age at disease onset was 46 years, and 79% of the patients were female. The numbers of PM, DM, and CADM cases were 40, 30, and 7, respectively. The median time between disease onset and starting treatment was 3 months. In addition, the median time between disease onset and the J-HAQ evaluation was 105 months. ILD, cardiomyopathy, and malignancy were found in 52 (68%), 13 (17%), and 3 (4%) patients, respectively. In regard to MSAs, anti-ARS, anti-MDA5, anti-Mi-2, anti-NXP-2, anti-SRP, and anti-TIF1-*γ* antibodies were detected in 22 (29%), 7 (9%), 2 (3%), 2 (3%), 9 (12%), and 4 (5%) patients, respectively. Disease relapse occurred in 30 (39%) patients.

### 3.2. Severity of Physical Dysfunction and Disease Activity in PM/DM Patients

**Figure 1:**
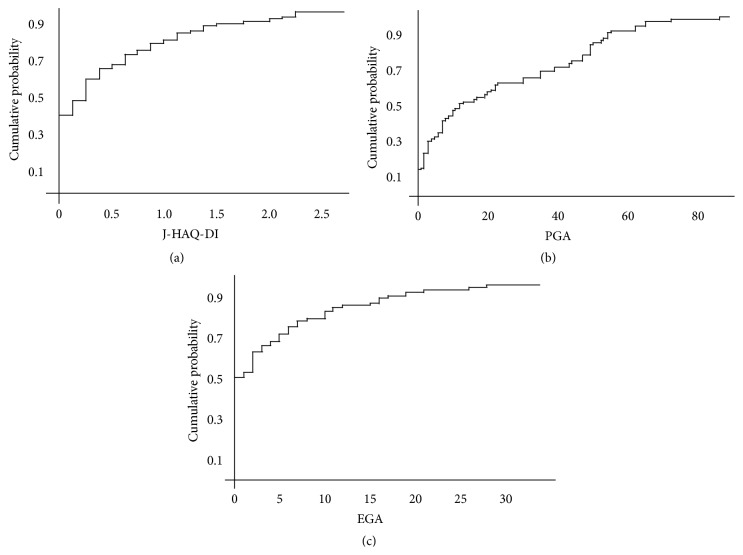
The J-HAQ-DI (a), PGA (b), and EGA (c) scores in enrolled PM/DM patients. Thirty percent of enrolled patients showed physical dysfunction (J-HAQ-DI > 0.5) (a). The PGA (b) and EGA (c) scores were less than 10 in 40% and 80% of patients, respectively. J-HAQ-DI: Japanese version of the Health Assessment Questionnaire Disability Index; PGA: Patient Global Assessment; EGA: Evaluator's Global Assessment.

Figure 1(a) presents the cumulative probability of physical dysfunction among the enrolled patients. The J-HAQ-DI score was greater than 0 and 0.5 in 57% and 30% of the patients, respectively. As shown in Figure 1(b), disease activity evaluated by patients was revealed in 60% of patients. In contrast, disease activity evaluated by physicians was revealed in 20% of patients (Figure 1(c)).

### 3.3. Associations between Physical Dysfunction and Disease Activity

**Figure 2:**
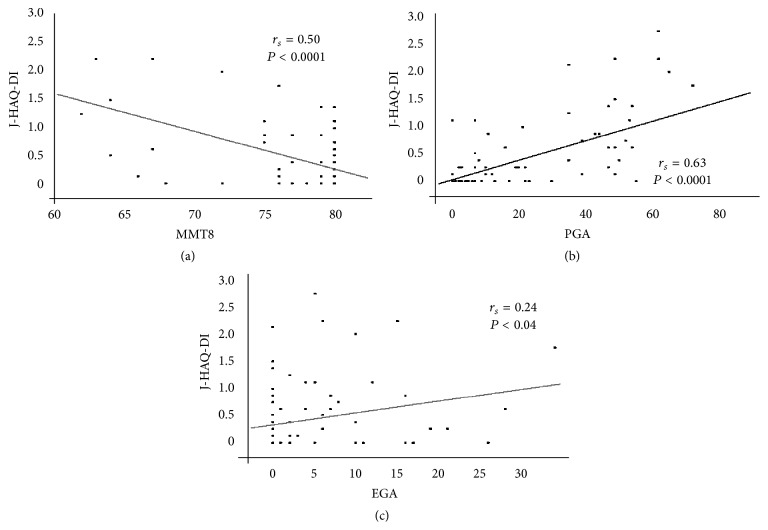
Correlations between the J-HAQ-DI score and the MMT8 (a), PGA (b), and EGA (c) scores. The J-HAQ-DI scores were significantly correlated with the MMT8 (a), PGA (b), and EGA (c) scores. Spearman's rank correlation coefficient (*r*_*s*_) was used for statistical analysis. J-HAQ-DI: Japanese version of the Health Assessment Questionnaire Disability Index; MMT: manual muscle test; PGA: Patient Global Assessment; EGA: Evaluator's Global Assessment.

Figure 2(a) presents the association between the J-HAQ-DI and MMT8 assessments. Physical dysfunction was significantly correlated (*r*_*s*_ = 0.50, *P* < 0.001) with muscle weakness. In addition, the severity of physical dysfunction was significantly correlated (*r*_*s*_ = 0.63, *P* < 0.0001) with the PGA results (Figure 2(b)). In contrast, the association between physical dysfunction and EGA results was weakly significant (*r*_*s*_ = 0.24, *P* = 0.04).

### 3.4. Clinical Manifestations Associated with Physical Dysfunction after Treatment

As shown in Table 2, we conducted a multivariate analysis to identify the clinical factors and complications associated with physical dysfunction after treatment in PM/DM patients. Physical dysfunction was defined as a J-HAQ-DI score of more than 0.5 [29]. Multivariate analysis found that age at disease onset (odds ratio (OR) = 1.07, *P* = 0.003), female gender (OR = 13.6, *P* = 0.0075), and CK levels before treatment (OR = 1.0006, *P* = 0.019) were significantly associated with physical dysfunction after treatment.

### 3.5. A Comparison of Autoantibodies between Patients with Normal and Dysfunctional Physical Functioning

To identify whether MSAs or MAAs are associated with physical dysfunction after treatment, we compared the positivity rates of each autoantibody between patients with normal and dysfunctional physical functioning. As shown in Table 3, the positivity rate for anti-SRP antibodies was significantly higher (*P* = 0.02) in the dysfunctional patients compared to normal patients. In contrast, the presence of anti-MDA5 antibodies was associated with normal physical function after treatment, although the relationship was not statistically significant.

**Figure 3:**
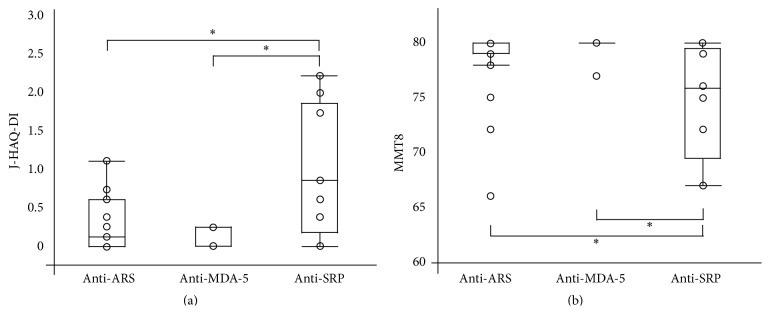
Associations between physical dysfunction and MSAs in PM/DM patients. The presence of anti-SRP antibodies was more significantly associated with severe physical dysfunction and muscle weakness after treatment. ^*∗*^ *P* value was less than 0.05. J-HAQ-DI: Japanese version of the Health Assessment Questionnaire Disability Index; ARS: aminoacyl-tRNA synthetase; MDA-5: melanoma differentiation-associated gene 5; SRP: signal recognition particle; MMT: manual muscle test.

Figure 3 presents a comparison of the J-HAQ-DI and MMT8 scores between patients with anti-ARS, anti-MDA5, or anti-SRP antibodies. Patients with anti-SRP antibodies showed significantly more severe physical dysfunction and muscle weakness than those with anti-ARS or anti-MDA5 antibodies. The median score of PGA was higher as 44 points in patients with anti-SRP antibodies than those without anti-SRP antibodies as 10 points, although the *P* value was 0.08, which could not reach statistical significance. In addition, the median score of EGA was less than 5 points in both these subsets. Therefore, these findings indicated that the severity of physical dysfunction could be attributed to disease damage rather than disease activity in myositis. On the other hand, there was no significant difference in the J-HAQ-DI and MMT8 scores between patients with anti-ARS antibodies and those with anti-MDA5 antibodies.

## 4. Discussion

The overall survival prognosis of PM/DM patients has recently improved, although some patients still suffer from physical dysfunction after treatment [5]. There have been few reports in the literature regarding the long-term physical function outcomes of PM/DM patients. Maugars et al. found that approximately 90% of PM/DM patients experienced muscular disability at a 3-year follow-up visit [30]. Ponyi et al. reported that, in 87 PM/DM patients, only 17.5% had no disability, and 12.5% were severely disabled; the remaining patients (70%) were mildly to moderately disabled [31]. In our present study, the median duration between disease onset and the evaluation of physical functioning was 105 months. Approximately 10 years had passed from the onset of PM/DM in most patients enrolled in this study. In 57% of the enrolled patients, the J-HAQ-DI score was greater than 0, which suggests that at least half of PM/DM patients had trouble in daily living activities, even 10 years after remission induction therapy. Thus, PM/DM continues to have a great impact on daily life in the long term.

In the present study, differences were observed between the evaluations of disease activity by patients and physicians. Although it may be difficult for patients to distinguish between irreversible damage and reversible inflammatory symptoms, IMACS defines active inflammation as disease activity. In our study, the age at disease onset, female gender, and CK levels before treatment were significant factors associated with the severity of the J-HAQ-DI scores. Clarke et al. assessed functional status in 257 PM/DM patients and found that physical dysfunction was associated with disease duration [5]. However, in our study, there was not a significant association between the J-HAQ-DI scores and disease duration. The median duration between disease onset and starting treatment was relatively short (3 months) in our patients, which may explain the difference between our findings and those of a previous report. In contrast, the age at disease onset and female gender were associated with physical dysfunction after treatment in the present study. Elderly patients and female patients have less muscle than younger patients and male patients; therefore, even after remission induction treatment, physical dysfunction may be sustained in elderly or female patients. CK serum levels before treatment were also associated with physical dysfunction after treatment in our study. Marie et al. reported that functional disability might be due to the adverse effects of corticosteroids, such as myopathy, osteoporosis, vertebral compression fracture, and avascular necrosis [6]. In our study, the CK levels before treatment may reflect the cumulative dosages of prednisolone (PSL) (the data could not be obtained). Physicians usually check the CK levels and decide whether the dosage of PSL could be tapered. Therefore, patients with lower CK levels before treatment may reach normal CK levels faster than those with higher CK levels and may be treated with smaller cumulative dosages of PSL. In addition, smaller cumulative dosages of PSL are associated with lower risks of the adverse effects of PSL, including steroid myopathy [32].

In the present study, with regard to the associations between MSAs and J-HAQ-DI scores, the presence of anti-SRP antibodies was more significantly associated with severe physical dysfunction after treatment compared to other MSAs (anti-ARS and anti-MDA5). In contrast, anti-MDA5 positivity was more strongly associated with better physical function after treatment compared to other MSAs. As far as we know, the present study is the first to report the association between autoantibodies and functional disability after treatment in PM/DM patients. Anti-SRP-associated myopathy is characterised by necrotising myopathy that is refractory and relapsing after immunosuppressive therapies. The patients of high CK levels or anti-SRP positive might show high disease activities at the onset of myositis, and their cumulative doses of oral glucocorticoid could be much more. The muscle weakness is caused not only by myositis itself, but also by steroid myopathy [33]. Moreover, our study demonstrated that PGA score was higher in patients with anti-SRP antibodies than others, although there was no difference of EGA score between the two subsets. There was a discrepancy between PGA score and EGA score in patients with anti-SRP antibodies. Therefore, the severity of physical dysfunction after treatment could be attributed to disease damage that resulted from previously active disease or from complications of therapy rather than ongoing disease activity in myositis. On the other hand, patients in East Asia with anti-MDA5 positivity showed complications of dermatitis and rapidly progressive- (RP-) ILD without myositis [9]. In addition, patients with anti-MDA5 antibodies usually show relatively mild dysfunction in daily life after RP-ILD improves, although some patients may succumb to respiratory failure caused by RP-ILD despite immunosuppressive therapies. Therefore, the measurement of MSAs is useful for predicting the physical function outcomes after treatment for PM/DM.

Several limitations of the present study should be considered. First, the patients were retrospectively enrolled. Second, the time between the onset of disease and the evaluation of disease status was different for each patient. In addition, disease status was only evaluated after treatment and not before treatment. Third, sera samples could not be obtained from all enrolled patients. Moreover, patients who died from malignancy, ILD, or other causes could not be included in the present study. Thus, selection bias may have been present in this study.

In conclusion, approximately one-half of PM/DM patients showed difficulties in activities of daily living, even after receiving remission induction therapy. The age at disease onset, gender, CK level before treatments, and anti-SRP positivity were significant factors associated with physical dysfunction after treatment in patients with PM/DM.

## Figures and Tables

**Table 1:** Clinical characteristics of the enrolled PM/DM patients.

	(*n* = 77)
Age at disease onset, median years	46 (37–58)
Female, number (%)	61 (79)
PM, DM, CADM, number	40, 30, 7
Duration from disease onset to initiation of treatment, median months	3 (2–8)
Duration from disease onset to enrolment in this study, median months	105 (46–143)
Complications at first visit, number (%)	
Interstitial lung disease	52 (68)
Cardiomyopathy	13 (17)
Malignancy	3 (4)
Myositis-specific autoantibodies, number (%)	
Anti-ARS	22 (29)
Anti-MDA5	7 (9)
Anti-Mi-2	2 (3)
Anti-NXP-2	2 (3)
Anti-SRP	9 (12)
Anti-TIF1-*γ*	4 (5)
Myositis-associated autoantibodies, number (%)	
Anti-Ku	3 (4)
Anti-U1-snRNP	10 (13)
Anti-SS-A	30 (39)
Treatment	
Initial dosage of PSL, median mg/day	50 (40–60)
Administration of immunosuppressant agents, number (%)	37 (48)
Presence of relapse, number (%)	30 (39)

Values represent medians (interquartile range). *P* values <0.05.

PM: polymyositis; DM: dermatomyositis; CADM: clinically amyopathic dermatomyositis; ARS: aminoacyl-tRNA synthetase; MDA5: melanoma differentiation-associated gene 5; NXP-2: nuclear matrix protein-2; SRP: signal recognition particle; TIF1-*γ*: transcriptional intermediary factor 1-*γ*; PSL: prednisolone.

**Table 2:** Clinical manifestations associated with physical dysfunction in PM/DM patients.

	Odds ratio (95% CI)^*∗*^	*P* value
Age at disease onset	1.07 (1.02–1.14)	0.0030
Female gender	13.6 (1.88–168.6)	0.0075
Duration from disease onset to the following:		
Initiation of treatment	1.01 (0.94–1.13)	0.45
Normalisation of CK level	1.008 (0.92–1.05)	0.80
Evaluation of the J-HAQ-DI score	1.009 (0.99–1.02)	0.15
Laboratory findings before initial treatment		
CK	1.0006 (1.0001–1.001)	0.019
LDH	0.99 (0.99–1.005)	0.10
CRP	0.87 (0.58–1.14)	0.35
Complications		
ILD	1.28 (0.31–5.60)	0.73
Cardiomyopathy	3.61 (0.63–22.9)	0.15
Malignancy	0.07 (0.0008–2.83)	0.16
Initial dosage of PSL	1.04 (0.98–1.12)	0.20
Administration of immunosuppressant agents	1.23 (0.28–5.45)	0.78
Presence of relapse	1.67 (0.42–6.75)	0.46

Statistical analyses were performed using multivariate analysis.

^*∗*^Odds ratio and confidential intervals were calculated per unit.

*P* values <0.05.

PM: polymyositis; DM: dermatomyositis; CK: creatine kinase; J-HAQ-DI: Japanese version of the Health Assessment Questionnaire Disability Index; LDH: lactate dehydrogenase; CRP: C-reactive protein; ILD: interstitial lung disease; PSL: prednisolone.

**Table 3:** A comparison of autoantibody profiles between PM/DM patients with and those without physical dysfunction.

	Physical normal function (*n* = 46)	Physical dysfunction (*n* = 21)	*P* value
Myositis-specific autoantibodies, number (%)			
Anti-ARS	16 (34)	6 (29)	0.62
Anti-MDA5	7 (15)	0 (0)	0.09
Anti-Mi-2	1 (2)	1 (5)	0.53
Anti-NXP-2	2 (4)	0 (0)	1.00
Anti-SRP	3 (7)	6 (29)	0.02
Anti-TIF1-*γ*	3 (7)	1 (5)	0.89
Myositis-associated autoantibodies, number (%)	7 (21)	6 (14)	0.38
Anti-Ku	3 (7)	0 (0)	0.55
Anti-U1-snRNP	6 (13)	4 (19)	0.71
Anti-SS-A	21 (46)	9 (43)	1.00

Physical dysfunction was defined as a J-HAQ-DI score greater than 0.5.

Statistical analyses were performed using the Mann-Whitney *U* test.

*P* values <0.05.

PM: polymyositis; DM: dermatomyositis; ARS: aminoacyl-tRNA synthetase; MDA5: melanoma differentiation-associated gene 5; NXP-2: nuclear matrix protein-2; SRP: signal recognition particle; TIF1-*γ*: transcriptional intermediary factor 1-*γ*.
